# Biological Response to Macroporous Chitosan-Agarose Bone Scaffolds Comprising Mg- and Zn-Doped Nano-Hydroxyapatite

**DOI:** 10.3390/ijms20153835

**Published:** 2019-08-06

**Authors:** Paulina Kazimierczak, Joanna Kolmas, Agata Przekora

**Affiliations:** 1 Department of Biochemistry and Biotechnology, Medical University of Lublin, Chodzki 1 Street, 20-093 Lublin, Poland; 2 Department of Analytical Chemistry and Biomaterials, Medical University of Warsaw, Banacha 1 Street, 02-097 Warsaw, Poland

**Keywords:** mesenchymal stem cells, osteoblasts, osteogenic differentiation, cell spreading, proliferation, tissue engineering

## Abstract

Modification of implantable scaffolds with magnesium and zinc for improvement of bone regeneration is a growing trend in the engineering of biomaterials. The aim of this study was to synthesize nano-hydroxyapatite substituted with magnesium (Mg^2+^) (HA-Mg) and zinc (Zn^2+^) (HA-Zn) ions in order to fabricate chitosan-agarose-hydroxyapatite (HA) scaffolds (chit/aga/HA) with improved biocompatibility. Fabricated biomaterials containing Mg^2+^ or Zn^2+^ were tested using osteoblasts and mesenchymal stem cells to determine the effect of incorporated metal ions on cell adhesion, spreading, proliferation, and osteogenic differentiation. The study was conducted in direct contact with the scaffolds (cells were seeded onto the biomaterials) and using fluid extracts of the materials. It demonstrated that incorporation of Mg^2+^ ions into chit/aga/HA structure increased spreading of the osteoblasts, promoted cell proliferation on the scaffold surface, and enhanced osteocalcin production by mesenchymal stem cells. Although biomaterial containing Zn^2+^ did not improve cell proliferation, it did enhance type I collagen production by mesenchymal stem cells and extracellular matrix mineralization as compared to cells cultured in a polystyrene well. Nevertheless, scaffolds made of pure HA gave better results than material with Zn^2+^. Results of the experiments clearly showed that modification of the chit/aga/HA scaffold with Zn^2+^ did not have any positive impact on cell behavior, whereas, incorporation of Mg^2+^ ions into its structure may significantly improve biocompatibility of the resultant material, increasing its potential in biomedical applications.

## 1. Introduction

In recent years, there has been growing interest in bone tissue engineering due to a high clinical demand for biocompatible bone scaffolds and novel biomaterials. A common approach to bone tissue engineering involves the development of three-dimensional porous scaffolds that support osteoblasts/osteoprogenitor cell adhesion, proliferation, and differentiation at the implantation site. Biomaterials are often composed of ceramics (calcium phosphates and bioglass) that imitate the inorganic part of a bone, i.e., hydroxyapatite (HA), polymers which mimic the flexible organic matrix of the bone, or their composites [1,2]. Among calcium phosphates, synthetic HA is the most widely used and is characterized by good osteoinductivity and bioactivity. Unfortunately, HA ceramic is often fragile and possesses weak mechanical properties [3]. Therefore, a combination of bioceramics with polymers is of great interest in the engineering of biomaterials as it results in better surgical handiness, mechanical properties, and degradability of the resultant biomaterial [1].

In an attempt to achieve successful bone tissue regeneration, scientists have been studying the role of small molecules (e.g., growth factors and cytokines) and metal ions in the regeneration process. As a consequence, modification of implantable scaffolds with metal or nonmetal (fluoride) ions is a growing trend in the engineering of biomaterials [2]. One of the commonly used methods to incorporate ions in a biomaterial structure is the substitution of HA or bioglass with various elements during their fabrication process, e.g., magnesium (Mg^2+^), zinc (Zn^2+^), cobalt (Co^2+^), lithium (Li^+^), strontium (Sr^2+^), copper (Cu^2+^), and fluoride (F^-^). Doping of different ions with bioceramics contributes to the improvement of biological properties of the resultant material, and thereby to the enhancement of regeneration of bone tissue within the implantable site. Moreover, the degree of ions loaded into the scaffolds and the controlled release of the loaded ions to the injured tissue are very significant for appropriate therapeutic effects [2,3].

Among all of the metal ions, Mg^2+^ and Zn^2+^ are most often used in the engineering of biomaterials to improve the osteoconductivity of the scaffolds. Mg^2+^ is a very abundant element in the human body, which is essential for many physiological functions, including bone metabolism. Mg^2+^-doped bioceramic has been shown to trigger accelerated bone tissue regeneration in vitro and in vivo [4]. Previous studies revealed that Mg^2+^ ions influence osteogenesis via enhancing osteogenic gene expression, i.e., osteocalcin (OC), runt-related transcription factor 2 (Runx-2), insulin-like growth factor 1 (IGF-1), and osseointegration in vivo [5]. It has also been shown that Mg^2+^ ions enhance the cell proliferation rate of human bone marrow-derived stem cells (BMDSCs), up-regulate expression of genes for collagen and vascular endothelial growth factor (VEGF), and stimulate bone extracellular matrix (ECM) mineralization [6]. Furthermore, substitution of bioceramics with Mg^2+^ supports cell adhesion and spreading [7].

Another essential trace element involved in bone metabolism is Zn^2+^, which regulates the expression of genes related to osteogenic differentiation, e.g., Runx-2, bone alkaline phosphatase (bALP), type I collagen (Col I), OC, and osteopontin [8]. It has also been demonstrated that Zn^2+^ ions enhance cell proliferation, bALP activity, and collagen synthesis in mouse calvarial preosteoblast cell line (MC3T3-E1) preosteoblasts [9]. Importantly, biomaterials containing Zn^2+^ not only promote bone formation and mineralization, but also inhibit osteoclasts-mediated bone resorption [8,10], have anti-inflammatory activity (through their ability to antagonize TNF-α) [4], and have antibacterial properties [2].

Considering the biological activities of Mg^2+^ and Zn^2+^ and their impact on the bone regeneration process, the modification of bone scaffolds with the above-mentioned metal ions is highly justified. The goal of this work was to synthesize HA nanopowders substituted with Mg^2+^ (HA-Mg) and Zn^2+^ (HA-Zn) ions in order to fabricate macroporous chitosan-agarose-HA scaffolds (chit/aga/HA) with improved biocompatibility. Moreover, it was hypothesized that the scaffold containing both Mg^2+^ and Zn^2+^ ions in its structure could exhibit a synergic effect on the biological response. Thus, biomaterial comprised of a mix of HA-Mg and HA-Zn was also fabricated. The scaffolds were prepared based on the procedure described in a Polish patent application no. P.426788 which used a gas-foaming agent in combination with the lyophilization process to produce material characterized by high, open and interconnected porosity. It is noteworthy that both the production method of the bone scaffolds and also their composition are innovative since there are no studies in the literature describing biomaterials made of a chitosan-agarose cryogel matrix reinforced with HA nanopowder. Scientific reports published by other authors present biomaterials for potential bone regenerations which were fabricated by applying completely different production methods and using only two of the mentioned components, e.g., scaffolds composed of only chitosan and hydroxyapatite [11,12], agarose and hydroxyapatite [13,14,15] or chitosan and agarose [16].

This study conducts a comprehensive analysis of biological responses and the production of bone scaffolds, with the aim of determining the effect of Mg^2+^ and Zn^2+^ incorporated into the structure of the biomaterials on osteoblast adhesion, spreading, and proliferation, as well as on osteogenic differentiation of mesenchymal stem cells. This research was conducted in direct contact with the scaffolds (cells were seeded onto the biomaterials) and using fluid extracts of the materials to assess the effect of the released ions on cell behavior.

## 2. Results and Discussion

### 2.1. Characterization of Calcium Phosphate Ceramics (NanoHA)

**Figure 1:**
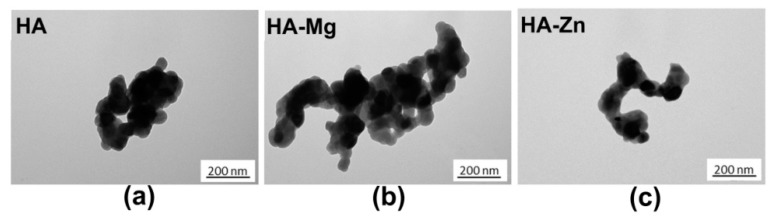
Transmission electron microscope (TEM) images of the obtained powders: (**a**) hydroxyapatite (HA), (**b**) HA substituted with magnesium (HA-Mg), and (**c**) HA substituted with zinc (HA-Zn) (scale bar = 200 nm).

All of the produced HA nanopowders were similar in size and morphology (Figure 1). The crystals were slightly irregular, plate-like, with an approximate diameter of 50–80 nm. Moreover, the crystals on the surface of all the samples tended to merge into dense clusters. It is important to note that the use of the wet precipitation method usually results in the formation of very fine apatite nanocrystals the size of 10–50 nm [17,18]. Moreover, the crystal shapes and sizes depend on many factors including the temperature and concentration of reagents, the addition of ion, and the temperature of calcination [19]. The obtained powders, after filtering and drying, were calcined at 1000 °C for 1 h in a furnace. The heating process resulted in crystals that were quite large and with rounded shapes.

**Figure 2:**
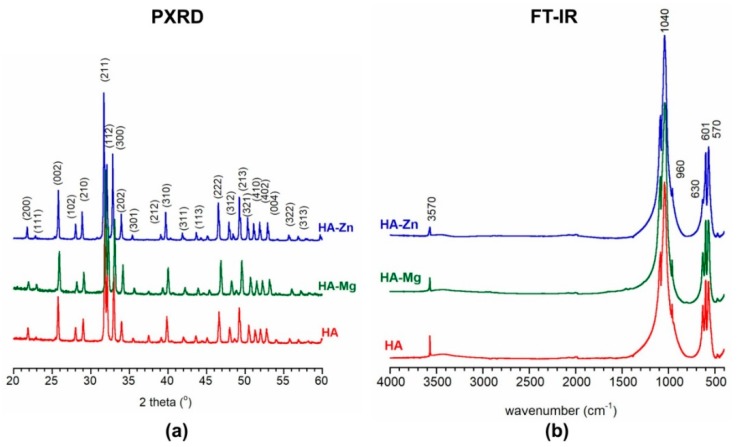
Powder X-ray diffraction (PXRD) patterns (**a**) and Fourier-transform infrared spectroscopy (FT-IR) spectra (**b**) of the synthesized calcium phosphate ceramics (nanoHA) powders.

The elemental analysis showed that the magnesium content in the HA-Mg sample was approximately 0.022 ± 0.002 mol and the zinc content in the HA-Zn sample was 0.028 ± 0.003 mol which is 73.3% and 93.3% of the assumed values, respectively. The powder X-ray diffraction (PXRD) patterns of HA, HA-Mg, and HA-Zn nanopowders are presented in Figure 2a. It is evident that all the materials were comprised of hydroxyapatite as one crystalline phase (all the reflections conforming well to the standard stoichiometric hydroxyapatite, JCPDS 09-0432). All the patterns exhibited narrow reflections related to highly crystalline materials [19,20]. It seems that the introduction of magnesium or zinc did not considerably affect the apatitic structure. The unit cell parameters and the calculated crystallite sizes obtained from PXRD measurements are presented in Table 1.

Fourier-transform infrared spectroscopy (FT-IR) spectra of the produced HA nanopowders are shown in Figure 2b. In the 1200–900 cm^−1^ and 603–500 cm^−1^ ranges, intense, narrow and well separated bands from orthophosphates are visible (ν_1_ + ν_3_ and ν_4_, respectively). These bands are characteristic of well crystalline hydroxyapatite [20]. Moreover, the bands at 3570 cm^−1^ and 633 cm^−1^ correspond to stretching and librational bands of structural hydroxyl (OH) groups, respectively. It should be noted that the relative intensity of OH bands was the lowest in the HA-Zn spectrum. This may indicate the lowest content of these groups in the structural OH groups columns. According to the literature, the mechanism of zinc substitution into the apatitic crystal lattice may be complex, i.e., Zn^2+^ may replace Ca^2+^ cations in the crystal core or may be introduced into the structural OH columns [21,22]. Thus, the relative decrease of the OH content may be related to the zinc insertion between two oxygen atoms and the release of H^+^ [21]:

Zn^2+^ + Ca_10_(PO_4_)_6_(OH)_2_ → ZnCa_10_(PO_4_)_6_O_2_ + 2H^+^

All the spectra were devoid of bands originating from vibrations of OH groups of water. This was due to the use of high temperature (1000 °C) for sample calcination after wet synthesis. For the same reason, there were no bands from carbonates (at approximately 1500–1400 cm^−1^ and 870 cm^−1^).

**Figure 3:**
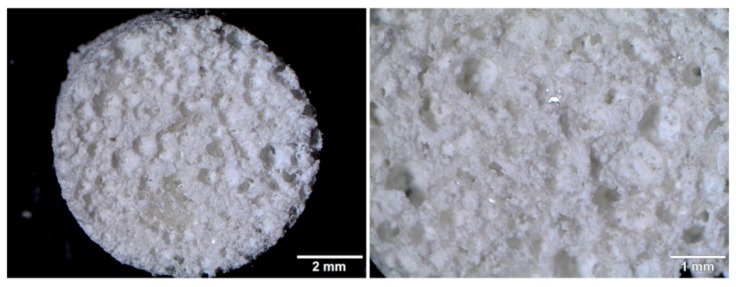
Microstructure of the chitosan-agarose-HA (chit/aga/HA) scaffold visualized by a stereoscopic microscope.

The following synthesized nanoHA powders were used for the production of the following highly macroporous bone scaffolds: chit/aga/HA, chit/aga/HA-Mg, chit/aga/HA-Zn, and chit/aga/HA-Mg/Zn which contained the 1:1 mixture of HA-Mg and HA-Zn. Figure 3 shows the sample stereoscopic microscope image of the produced biomaterial. There were no significant differences in microstructure and topography between respective scaffolds comprising metal-doped HA. The surface of all scaffolds was characterized by high roughness and macroporosity.

### 2.2. Evaluation of Ions Concentration in Scaffolds Extracts

The Mg^2+^ and Zn^2+^ ion concentrations in the prepared scaffold extracts are shown in Table 2. The chit/aga/HA (control material) and chit/aga/HA-Zn scaffolds exhibited a slight uptake of Mg^2+^ ions from the culture medium after 24 h incubation at 37 °C, whereas, chit/aga/HA-Mg and chit/aga/HA-Mg/Zn scaffolds released Mg^2+^ ions. Chit/aga/HA-Zn and chit/aga/HA-Mg/Zn samples caused an increase in the Zn^2+^ ion concentration in the medium, however chit/aga/HA-Zn released significantly more ions as compared to the chit/aga/HA-Mg/Zn.

### 2.3. In Vitro Cell Culture Experiments

#### 2.3.1. Cytotoxicity Assessment

**Figure 4:**
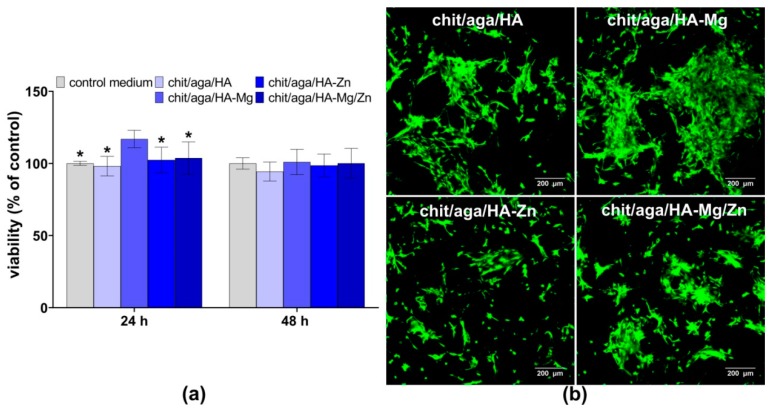
Cytotoxicity assessment of the scaffolds against mouse calvarial preosteoblast cell line (MC3T3-E1) cells: (**a**) MTT assay performed using scaffolds extracts (*statistically significant results as compared to chit/aga/HA-Mg extract, *P* < 0.05, one-way ANOVA followed by Tukey’s test); (**b**) confocal laser scanning microscope (CLSM) images presenting live-dead staining (viable cells, green fluorescence and dead cells, red fluorescence) of MC3T3-E1 cells cultured for 48 h on the surface of the scaffolds; magnified 100×, scale bar = 200 µm.

The MTT test showed that scaffolds were nontoxic to MC3T3-E1 osteoblasts (Figure 4a). After 24 h exposure time, the chit/aga/HA-Mg extract significantly increased cell metabolism as compared to other extracts and the control medium (*P* < 0.0001 as compared to the control medium and extracts of chit/aga/HA and chit/aga/HA-Zn, *P* = 0.0004 as compared to chit/aga/HA-Mg/Zn extract). This is in agreement with studies performed by He et al. that revealed the ability of Mg^2+^ ions to increase osteoblasts viability via enhancing gap junction intercellular communication between the cells which are responsible for transmitting signals [23]. Whereas, after 48 h exposure time, the viability of cells was near 100% for all investigated extracts (no statistically significant results were observed). Live-dead staining of MC3T3-E1 osteoblasts cultured on the surface of the biomaterials for 48 h confirmed nontoxicity of the scaffolds (Figure 4b).

Confocal laser scanning microscope (CLSM) images presented a great number of viable cells (green fluorescence), which were flattened and well attached to all scaffolds, confirming their good adhesion. Interestingly, the number of osteoblasts attached to the chit/aga/HA-Mg scaffold was noticeably higher as compared to other scaffolds. Thus, the tendency in both experiments (i.e., the indirect cytotoxicity test with the use of scaffolds extracts and the cytotoxicity test in direct contact with the biomaterials) was the same since the bone scaffold comprising HA-Mg, as well as extract enriched with Mg ions, had a positive effect on cell viability.

#### 2.3.2. Cell Adhesion and Spreading

**Figure 5:**
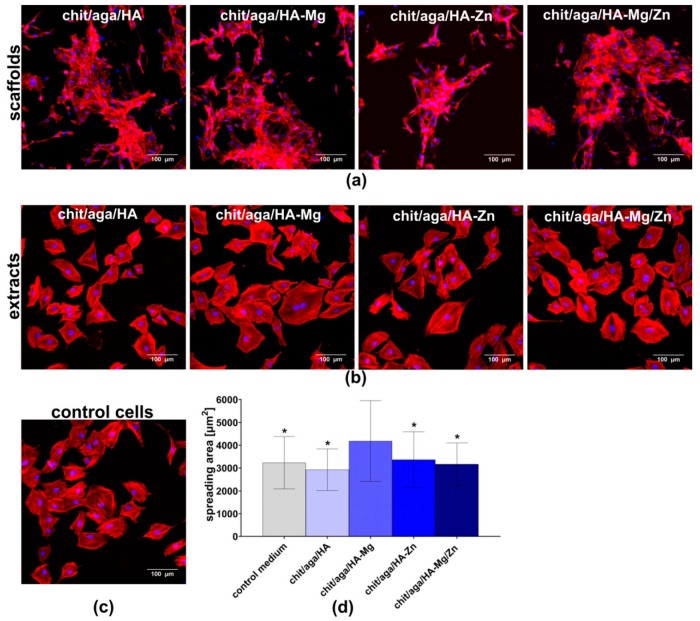
Evaluation of cell adhesion and spreading of MC3T3-E1 cells: (**a**) fluorescent staining of cell cytoskeleton when osteoblasts were cultured directly on the scaffolds; (**b**) fluorescent staining of cell cytoskeleton when osteoblasts were cultured in PS wells in the presence of scaffolds extracts (F-actin filaments, red fluorescence and nuclei, blue fluorescence, magnified 200×, scale bar = 100 µm); (**c**) morphology of control cells maintained in culture medium and grown in the polystyrene wells PS wells; and (**d**) quantitative evaluation of spreading area of cells cultured in the PS wells in the presence of scaffolds extracts (* statistically significant results as compared to chit/aga/HA-Mg extract, *P* < 0.05, one-way ANOVA followed by Tukey’s test).

The CLSM images showed that cells cultured on the biomaterials, as well as in the polystyrene wells (PS wells), in the presence of scaffolds extracts were well spread and had flattened morphology. Nevertheless, differences in the shape of the osteoblasts were observed between the cells cultured directly on the scaffolds and in the PS wells, which resulted from porous microstructure and roughness of the surface of the biomaterials (Figure 5a,b). Since the MC3T3-E1 cells formed clusters on the surface of the samples, quantitative evaluation of the cell spreading area was possible only for osteoblasts cultured in PS wells in the presence of the extracts.

Cells exhibited similar morphology when cultured in all extracts, however, osteoblasts cultured in the presence of chit/aga/HA-Mg extract revealed significantly higher (*P* < 0.0001) spreading area as compared to the cells maintained in other extracts and the control medium (Figure 5d), which was also confirmed by CLSM visualization (Figure 5b). However, such results were not observed for chit/aga/HA-Mg/Zn scaffold, although it contained Mg^2+^ ions in its structure. Nevertheless, it should be noted that chit/aga/HA-Mg/Zn was made of 15 wt% Mg-doped HA and 15 wt% Zn-doped HA, whereas, chit/aga/HA-Mg contained 30 wt% HA-Mg. According to the available literature, Mg-doped bioceramics has been proven to support cell adhesion and spreading on the biomaterial surface, which is mediated by membrane-associated adhesion receptors, i.e., integrins. The extracellular domain of integrins contains motifs which bond divalent cations (Mg^2+^ and Ca^2+^). The changes in extracellular concentration of these ions may alter the affinity of integrins to their respective ligands, and therefore affect cell adhesion [7].

#### 2.3.3. Cell Proliferation

Studies have reported that certain concentrations of Mg^2+^ and Zn^2+^ ions act as positive stimuli, enhancing osteoblasts proliferation and osteogenic differentiation [6,24,25,26]. Thus, it has been observed that researchers more often modify implantable biomaterials with the above-mentioned metal ions in order to improve biocompatibility of the resultant material.

**Figure 6:**
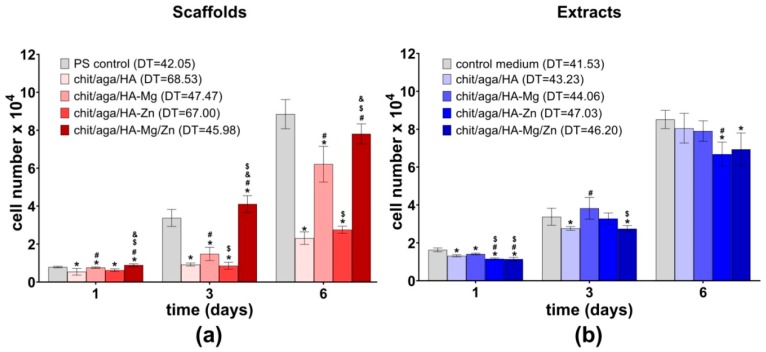
MC3T3-E1 cell proliferation analysis: (**a**) osteoblasts cultured on the surface of the scaffolds (PS control, cells cultured in the PS well of 96-multiwell plate) and (**b**) osteoblasts cultured in the PS wells in the presence of scaffolds extracts (control medium, cells maintained in culture medium). DT, doubling time in hours; * statistically significant results as compared to control; ^#^ statistically significant results as compared to chit/aga/HA; ^$^ statistically significant results as compared to chit/aga/HA-Mg; ^&^ statistically significant results as compared to chit/aga/HA-Zn; *P* < 0.05, one-way ANOVA followed by Tukey’s test.

In this study, osteoblasts proliferation was determined by evaluating the cell number on the surface of the biomaterials and in the PS wells upon exposure to the scaffold extracts after one, three, and six days of culture. Among all the fabricated samples, the biomaterials containing Mg-doped HA (chit/aga/HA-Mg and chit/aga/HA-Mg/Zn) were the most favorable to cell proliferation (Figure 6a). The MC3T3-E1 osteoblasts revealed significantly enhanced proliferation when cultured on these scaffolds as compared to the control material (chit/aga/HA). Importantly, the cells grown on the biomaterials containing HA-Mg revealed similar doubling time (DT) (i.e., defined as the period of time in hours required to double cell population) as compared to the control cells cultured in the PS well. Moreover, on the third day of the experiment, the number of cells on the surface of chit/aga/HA-Mg/Zn was significantly higher (*P* = 0.0372) as compared to the PS control. Interestingly, although Zn^2+^ was reported to enhance cell proliferation [27], incorporation of Zn^2+^ ions into the scaffold structure (chit/aga/HA-Zn) did not improve cell proliferation as compared to the control chit/aga/HA material. Thus, enhanced cell proliferation on the surface of the chit/aga/HA-Mg and chit/aga/HA-Mg/Zn scaffolds resulted from the presence of Mg^2+^ ions in their structure.

The experiment with the use of scaffold extracts showed negligible differences in cell proliferation between tested extracts of the materials (Figure 6b). Nevertheless, on the sixth day of the experiment, extracts of chit/aga/HA-Zn and chit/aga/HA-Mg/Zn significantly reduced the cell number as compared to the control cells maintained in the cultured medium. The DT values calculated for the MC3T3-E1 cells cultured in the presence of the extracts were comparable to the DT values recorded for the control cells. However, higher values of DT were observed for extracts containing Zn ions.

Observed divergent results that were obtained with direct contact and indirect experiments most likely resulted from various exposure times of scaffolds to the culture medium. The extraction process of the biomaterials lasted 24 h, whereas, during direct contact test, the scaffolds were incubated in culture medium for six days which allowed higher concentrations of released ions to be achieved. Importantly, our results contradict reports presented by other authors based on the available literature, that both Zn^2+^ and Mg^2+^ ions have the ability to stimulate proliferation of the cells [6,27]. Yoshizawa et al. [6] demonstrated that Mg^2+^ ions enhance proliferation of BMDSCs and their osteogenic differentiation. Whereas, Moon et al. [28] revealed that Zn^2+^ increased adipose tissue-derived mesenchymal stem cells (ADSCs) proliferation via activation of the ERK1/2 pathway. Similarly, Ullah et al. [29] showed that the incorporation of zinc oxide into chitosan-collagen scaffolds supports fibroblasts and keratinocytes adhesion, proliferation, and infiltration of biomaterial.

#### 2.3.4. Evaluation of Osteogenic Differentiation

Mesenchymal stem cells (MSCs) derived from bone marrow or adipose tissue are excellent biological sources of osteoprogenitor cells for bone tissue engineering applications. Detection of osteogenic differentiation markers in MSCs cultured on the surface of the biomaterial is undoubtedly proof of osteoconductive or osteoinductive (if the cells are cultured without osteogenic inducers) properties of the tested scaffold. During osteogenic differentiation, osteoprogenitor cells/osteoblasts produce several specialized proteins, which are considered as markers of the bone formation process. The differentiation process involves the following three main stages: (1) proliferation phase when the cells proliferate intensively and produce great amounts of Col I and fibronectin; (2) ECM synthesis phase when the cells reveal the highest bALP activity and intensively produce proteins of bone ECM; and (3) ECM mineralization phase when the cells exhibit moderate bALP production, high mineralization activity, and produce significant amounts of OC and osteopontin [30,31].

**Figure 7:**
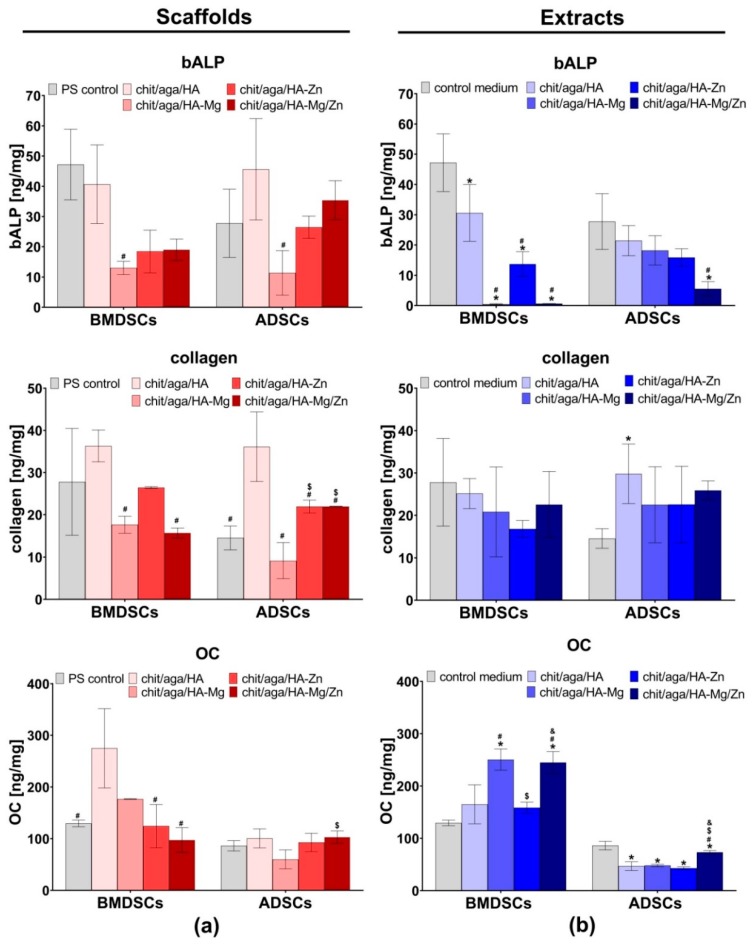
The level of osteogenic markers assessed by enzyme-linked immunosorbent assay (ELISAs): (**a**) level of markers in mesenchymal stem cells (MSCs) grown on the scaffolds (PS control, cells cultured in PS wells); (**b**) level of markers in MSCs cultured in PS wells in the presence of scaffolds extracts (control medium, cells maintained in osteogenic medium). The results were expressed as ng of marker per mg of total cellular proteins. * statistically significant results as compared to PS control/control medium, ^#^ statistically significant results as compared to chit/aga/HA, ^$^ statistically significant results as compared to chit/aga/HA-Mg, ^&^ statistically significant results as compared to chit/aga/HA-Zn, *P* < 0.05, one-way ANOVA followed by Tukey’s test.

In this study, the level of typical osteogenic markers (bALP, Col I, and OC) produced by MCSs grown on the surface of the biomaterials and in PS wells upon exposure to the scaffolds extracts was evaluated after 21 day culture. It was observed that BMDSCs and ADSCs cultured on the chit/aga/HA surface produced significantly higher amounts of bALP, Col I, and OC as compared to the cells cultured on other scaffolds (Figure 7a). Interestingly, MSCs cultured on the biomaterial containing Zn-doped HA (chit/aga/HA-Zn and chit/aga/HA-Mg/Zn) showed higher levels of bALP and Col I than that of cells cultured on the chit/aga/HA-Mg. Nevertheless, BMDSCs cultured on the chit/aga/HA-Mg showed higher OC synthesis as compared to chit/aga/HA-Zn and chit/aga/HA-Mg/Zn. Importantly, MSCs cultured on the surface of the scaffolds containing Mg-doped HA and Zn-doped HA did not exhibit enhanced synthesis of osteogenic markers as compared to the cells grown on biomaterial made of pure HA. Thus, the results of this study are not consistent with the information found in the available literature which reports an increased production of osteogenic markers by MSCs in the presence Mg^2+^ and Zn^2+^ ions [10,24,25,26,28,32]. However, it should be noted that differentiation of MSCs cultured on the surface of biomaterials may be affected by various factors such as chemical composition of the scaffolds, porosity, surface roughness [30,33], and even stiffness (Young’s modulus) of the materials [34,35]. It is well known that calcium phosphate-based materials with highly macroporous structures and rough surfaces may have the ability to induce osteogenic differentiation in MSCs [30]. Moreover, it has been demonstrated that cells cultured on substrates with low stiffness show enhanced osteogenic differentiation [35]. The scaffolds produced in this study that were made of calcium phosphate ceramics (nanoHA), showed high elasticity (low stiffness), and had a highly macroporous and rough structure (Figure 3). Therefore, the MSC differentiation observed on the surface of the investigated bone scaffolds was not only affected by Mg^2+^ and Zn^2+^ ions, but it was also a result of the microstructural, mechanical, and physicochemical properties of the materials. This may explain the discrepancy between the results obtained here and the results presented by other authors who performed studies on scaffolds having different chemical compositions and microstructural properties. Other factors which most likely influenced our results, as compared to other authors, were the divergent degree of ceramic substitution with Mg^2+^ and Zn^2+^ and the different release rates of these ions from the biomaterials.

**Figure 8:**
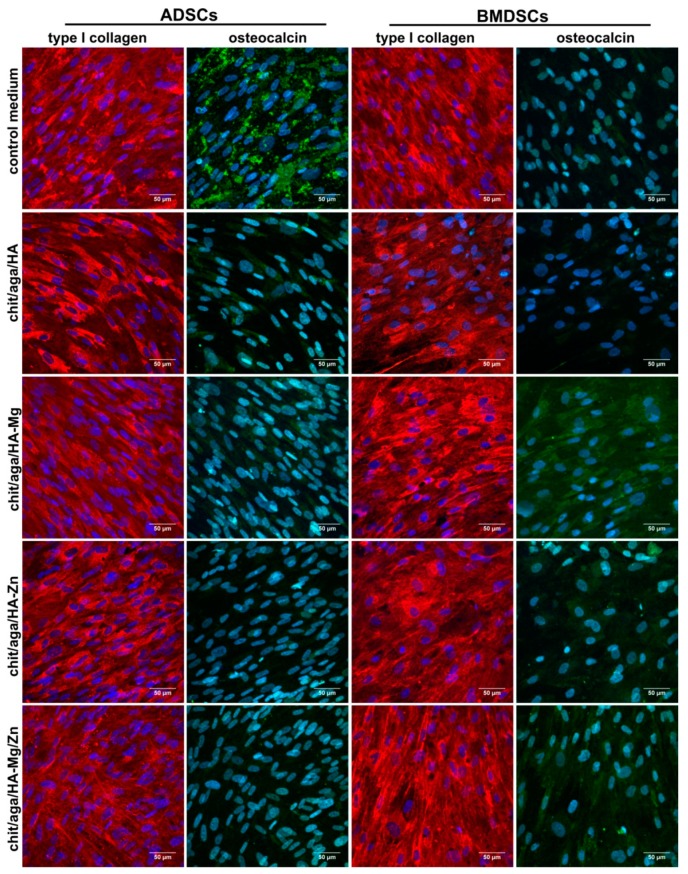
CLSM images presenting immunofluorescent (IF) staining of type I collagen and osteocalcin in the extracellular matrix (ECM) of MSCs cultured in PS wells in the presence of scaffolds extracts (control medium, cells maintained in osteogenic medium); collagen, red fluorescence; osteocalcin, green fluorescence; and nuclei, blue fluorescence, magnified 400×; scale bar = 50 µm.

**Figure 9:**
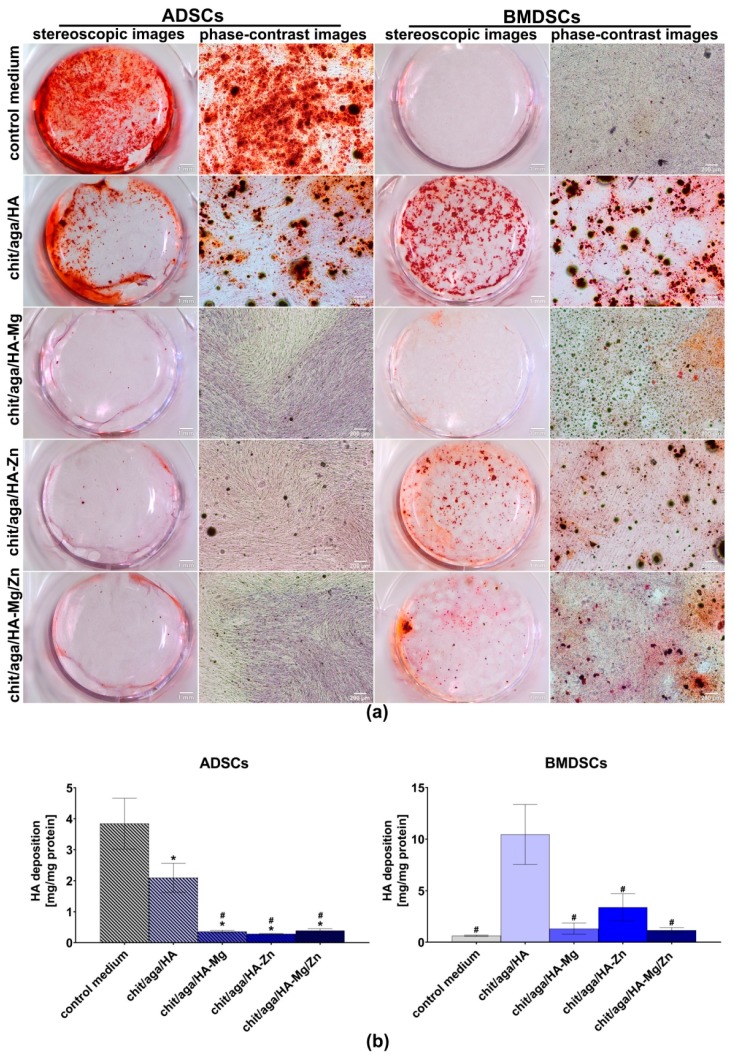
Evaluation of mineral deposition in ECM of MSCs cultured in PS wells in the presence of scaffolds extracts (control medium, cells maintained in osteogenic medium): (**a**) stereoscopic and phase-contrast images presenting ARS staining of bone marrow-derived stem cells (BMDSCs) and adipose tissue-derived mesenchymal stem cells (ADSCs) (stereoscopic images, scale bar = 1 mm and phase-contrast images, scale bar = 200 µm); (**b**) quantitative evaluation of mineral deposits in ECM of MSCs. The mineral deposition was expressed as mg of mineral per mg of total cellular proteins. * statistically significant results as compared to control medium, ^#^ statistically significant results as compared to chit/aga/HA, *P* < 0.05, one-way ANOVA followed by Tukey’s test.

In the case of the indirect test with the use of extracts of the scaffolds, osteogenic markers were detected by the enzyme-linked immunosorbent assays (ELISAs), and also by the immunofluorescent (IF) staining of Col I and OC, as well as by Alizarin Red S staining (ARS) of mineral deposited in ECM. The BMDSCs cultured in the control medium revealed significantly higher (*P* < 0.0001 as compared to the extracts of chit/aga/HA-Mg, chit/aga/HA-Zn, and chit/aga/HA-Mg/Zn; *P* = 0.0144 as compared to chit/aga/HA extract) bALP production as compared to the cells maintained in the extracts of the scaffolds. The very high level of bALP in the BMDSCs maintained in the control medium along with the decreased OC production (Figure 7b and Figure 8) and poor mineralization activity (Figure 9) may suggest that on the twenty-first day of the experiment these cells were still in the second stage of osteogenic differentiation (ECM synthesis). On the other hand, the BMDSCs exposed to the extracts of chit/aga/HA-Mg and chit/aga/HA-Mg/Zn scaffolds showed significantly reduced production of bALP, increased synthesis of OC which was also confirmed by IF staining (Figure 7b and Figure 8), and poor mineralization activity (Figure 9). Thus, despite a high amount of OC (protein which is related to ECM calcification [31]) in the ECM of BMDSCs, the mineralization process did not begin. These observations indicate that the biomaterial containing Mg^2+^ ions caused a significant reduction in bALP synthesis (enzyme essential for mineralization process [31]), delaying the third stage of osteogenic differentiation and ECM calcification. Interestingly, the BMDSCs cultured in the presence of the chit/aga/HA extract revealed a moderate level of bALP which is typical of the third stage of osteogenic differentiation. As a consequence, ARS staining demonstrated the highest mineralization activity of these cells (Figure 9). The positive impact of scaffold extracts (especially chit/aga/HA extract) on ECM mineralization (Figure 9) can be explained by the dissolution of the bioceramics and the stimulation of HA deposition by released (Ca^2+^) and phosphate (PO_4_^3−^) ions, which play an essential role in bone metabolism. Ca^2+^ is known to support osteoblast adhesion, proliferation, differentiation, and ECM mineralization. Moreover, it stimulates Ca-sensing receptors in osteoblasts and enhances expression of insulin-like growth factors, e.g., IGF-1 and IGF-2 [4,36]. Whereas, PO_4_^3−^ regulates cell proliferation and stimulates the expression of matrix Gla protein (MGP) and ECM mineralization by increasing IGF-1 synthesis [4,36,37].

In the case of ADSCs, all extracts of the biomaterials decreased bALP and OC synthesis, but increased Col I production as compared to the control cells maintained in the osteogenic medium (Figure 7b). Interestingly, the ADSCs maintained in the extracts of the scaffolds containing Mg^2+^ and/or Zn^2+^ ions showed significantly reduced mineralization activity as compared to the control cells and also as compared to the chit/aga/HA extract (Figure 9). The reduced level of bALP and OC, along with poor ECM calcification, suggests that ADSCs cultured in the extracts containing Mg^2+^ and/or Zn^2+^ were still in the first stage of osteogenic differentiation. Unlike cells exposed to the scaffold extracts, the ADSCs cultured in the control medium showed a moderate bALP level (Figure 7b), high OC production, and high mineralization activity (Figure 8 and Figure 9), and therefore they were in the third phase of osteogenic differentiation.

The results of this study demonstrated that the incorporation of Mg^2+^ and Zn^2+^ metal ions in the structure of the chit/aga/HA scaffold retard osteogenic differentiation of MSCs because stem cells cultured on the biomaterial made of pure HA or in the chit/aga/HA extract showed a higher level of osteogenic markers and higher mineralization activity as compared to other scaffolds. Therefore, modification of chit/aga/HA biomaterial with metal ions did not produce the expected results despite numerous reports about the positive impact of Mg^2+^ and Zn^2+^ ions on the bone formation process. Jin et al. [32] revealed that Zn^2+^ ions stimulated bALP activity, collagen secretion, and ECM mineralization in rat MSCs. Kim et al. showed that extract containing Mg^2+^ increased bALP activity and ECM mineralization in hFOB 1.19 cells [38]. Whereas, Thian et al. demonstrated enhanced proliferation and differentiation of ADSCs cultured on biomaterial made of Zn-doped HA [27].

Nevertheless, the literature has reported other biological activities of metal ions such as the improvement of cell adhesion, spreading, and proliferation, which were also found in our results. This study demonstrated that the incorporation of Mg^2+^ ions in the chit/aga/HA structure increased the spreading of the osteoblasts and promoted cell proliferation on the scaffold surface. Importantly, although, in general, Mg^2+^ ions retarded osteogenic differentiation of MSCs, the presence of Mg^2+^ in the extracts of biomaterials enhanced osteocalcin production by BMDSCS as compared to the chit/aga/HA extract and the control medium. Surprisingly, Zn^2+^ incorporation into the chit/aga/HA scaffold did not improve cell spreading and proliferation, but MSCs cultured on the scaffolds or in extracts containing Zn^2+^ showed enhanced Col I production and extracellular matrix mineralization as compared to the cells cultured in the PS well. However, biomaterial made of pure HA gave better results than material with Zn^2+^ incorporation.

In conclusion, the results of this study showed that modification of chit/aga/HA scaffold with Zn^2+^ does not have any positive impact on cell behavior, whereas, incorporation of Mg^2+^ ions in its structure may significantly improve the biocompatibility of the resultant material, increasing its potential in biomedical applications. Interestingly, contrary to the common opinion that hydroxyapatite substituted with Zn ions may significantly improve cell proliferation and differentiation, we showed that Zn-doped HA was less efficient than Mg-doped HA and even worsened the biocompatibility of the resulting bioceramic-based material as compared to the scaffold containing pure HA. Thus, our results provide new insight into the use of Zn ions for scaffold modifications in the engineering of biomaterials.

## 3. Materials and Methods

### 3.1. Synthesis and Characterization of NanoHA

The synthesis of three nanopowders: undoped, Mg, and Zn which contained hydroxyapatite (HA, HA-Mg, and HA-Zn) were prepared according to the wet precipitation method. Calcium nitrate tetrahydrate (Ca(NO_3_)_2_·4H_2_O), diammonium hydrogen phosphate ((NH_4_)_2_HPO_4_), zinc nitrate hexahydrate (Mg(NO_3_)_2_·6H_2_O) and magnesium nitrate hexahydrate (Zn(NO_3_)_2_·6H_2_O) were purchased from Sigma-Aldrich Chemicals (Warsaw, Poland) and used as sources of calcium, phosphorus, magnesium, and zinc, respectively. The expected doping levels for Mg^2+^ or Zn^2+^ were 0.03 mol for 1 mol of HA-Mg or HA-Zn powder, respectively. The aqueous solutions of the precursors were prepared in such a way that the Ca/P in HA, (Ca+Mg)/P in HA-Mg, and (Ca+Zn)/P in HA-Zn molar ratio was equal to 1.67. The powders were obtained via a standard precipitation method by slowly adding an aqueous solution of (NH_4_)_2_HPO_4_, to mixed aqueous solutions of Ca(NO_3_)_2_·4H_2_O (and Mg(NO_3_)_2_·6H_2_O or Zn(NO_3_)_2_·6H_2_O) under vigorous stirring. The pH of the mixtures was maintained at 9 by adding ammonia solution (Avantor Performance Materials, Gliwice, Poland). The obtained suspensions were heated at 70 °C for 2 h and then kept in static conditions for aging. After 24 h, the precipitates were filtered, washed several times, and dried at 100 °C. Then, the powders were calcined at 1000 °C for 1 h.

The produced nanoHA powders were subjected to structural and physicochemical analysis to determine the Zn or Mg content, crystallinity, and the size and shape of the particles. Transmission electron microscope (TEM) (JEM 1400, Jeol Co., Tokyo, Japan) studies were carried out at an accelerating voltage of 80 kV. The analyzed powders were prepared by dispersing a sample in 96° ethanol, followed by dropping it into a copper grid covered with Formvar film, and air drying. Elemental analysis of the doped cations (Mg^2+^ and Zn^2+^) were performed using inductively coupled plasma spectrometry (ICP-OES, Thermo Scientific iCAP 7400 Duo, Waltham, MA, USA). For this purpose, the powders were dissolved in supra pure 63% nitric acid (Sigma-Aldrich Chemicals, Warsaw, Poland) and deionized water. The PXRD patterns were recorded on a Bruker DX8 Discover diffractometer (CuKα radiation, λ = 1.54 Å) and registered in the 2-theta range within 20° to 60°. Both *a* and *c* lattice parameters of the unit cell were obtained from the TOPAS program. The software uses Rietveld refinement based on analytical profile functions and least squares algorithms to fit a theoretical to a measured PXRD. Moreover, the software uses Scherrer formula for crystallite size calculation. The FT-IR transmission spectra were collected in the range of 4000–400 cm^−1^, at room temperature on a Perkin Elmer Spectrum 1000 spectrometer with a resolution of 2 cm^−1^ and 30 scans.

### 3.2. Fabrication of the Scaffolds

Four types of chitosan-agarose-hydroxyapatite scaffolds containing 30 wt% pure HA (control sample marked as chit/aga/HA), 30 wt% Mg-doped HA (marked as chit/aga/HA-Mg), 30 wt% Zn-doped HA (marked as chit/aga/HA-Zn), and mixture of 15 wt% Mg-doped HA and 15 wt% Zn-doped HA (marked as chit/aga/HA-Mg/Zn) were synthesized in accordance with the procedure described in the Polish patent application no. P.426788. The scaffolds were produced by a combination of gas-foaming and freeze-drying methods. In brief, composites were prepared by mixing suspension of 2 wt% chitosan (50–190 kDa molecular weight, 75%–85% deacetylation degree, viscosity ≤300 cP, Sigma-Aldrich Chemicals, Warsaw, Poland) and 5 wt% agarose (low EEO, gel point 36 ± 1.5 °C, Sigma-Aldrich Chemicals) in 2% acetic acid solution (Avantor Performance Materials, Gliwice, Poland) with the appropriate type of HA and sodium bicarbonate (NaHCO_3_, Sigma-Aldrich Chemicals, Warsaw, Poland) added as a foaming agent. The obtained paste was put in a cylinder-shaped vial, then heated in a water bath at a temperature of 95 °C, cooled, frozen in a liquid nitrogen vapor phase, and subjected to freeze drying (LYO GT2-Basic, SRK Systemtechnik GmbH, Riedstadt, Germany). The final samples were neutralized in 1% sodium hydroxide solution (NaOH, Avantor Performance Materials, Gliwice, Poland), rinsed with deionized water, and left to air dry. The microstructure of the produced scaffolds was visualized by a stereoscopic microscope (Olympus SZ61TR, Olympus Polska Sp. z o. o., Warsaw, Poland). Prior to all experiments, the samples were sterilized by ethylene oxide. Before the cell culture experiments, the samples were preincubated overnight in appropriate complete culture medium at 37 °C.

### 3.3. Preparation of Scaffolds Extracts

Extracts of the scaffolds were prepared in accordance with ISO 10993-12 standard. Briefly, the sterile scaffolds were immersed in an appropriate complete culture medium (maintaining the ratio of 100 mg sample per 1 mL medium) and incubated at 37 °C for 24 h. The culture medium incubated without scaffolds served as the control (marked as control). The Mg^2+^ and Zn^2+^ ions concentrations in the collected extracts were evaluated by colorimetric method using commercially available kits for the determination of magnesium (BioMaxima, Lublin, Poland) and zinc ions (Sigma-Aldrich Chemicals, Warsaw, Poland). The prepared extracts were subjected to in vitro cell culture experiments.

### 3.4. In Vitro Cell Culture Experiments

Mouse calvarial preosteoblast cell line (MC3T3-E1 Subclone 4, ATCC-LGC standards, Teddington, UK), human bone marrow-derived mesenchymal stem cells (BMDSCs, ATCC-LGC standards, Teddington, UK), and human adipose tissue-derived mesenchymal stem cells (ADSCs, ATCC-LGC standards, Teddington, UK) were used in the experiments. The MC3T3-E1 cells were cultured in an alpha-MEM medium (Gibco, Life Technologies, Carlsbad, CA, USA) containing 10% fetal bovine serum (FBS, Pan-Biotech GmbH, Aidenbach, Bavaria, Germany), 100 U/mL penicillin, 100 μg/mL streptomycin (Sigma-Aldrich Chemicals, Warsaw, Poland). The BMDSCs were cultured in Mesenchymal Stem Cell Basal Medium (ATCC-LGC Standards, Teddington, UK) supplemented with the components of Bone Marrow-Mesenchymal Stem Cell Growth Kit (ATCC-LGC Standards, Teddington, UK), and antibiotics: 10 U/mL penicillin and 10 μg/mL streptomycin. The ADSCs were cultured in Mesenchymal Stem Cell Basal Medium supplemented with the components of Adipose-derived Mesenchymal Stem Cell Growth Kit Low Serum (ATCC-LGC Standards, Teddington, UK), and antibiotics: 10 U/mL penicillin and 10 μg/mL streptomycin. All cells were incubated at 37 °C in a humidified atmosphere of 5% CO_2_ in air atmosphere.

#### 3.4.1. Cytotoxicity Assessment

Cytotoxicity assessment was carried out according to ISO 10993-5 standard by indirect method using 24 h extracts of the samples prepared as described in Section 3.3. The MC3T3-E1 cells were seeded into 96-multiwell plate in 100 µL of a complete culture medium at a concentration of 2 × 10^4^ cells per well. After 24 h incubation, the culture medium was discarded, and appropriate scaffolds extracts were added. Culture medium served as a negative control of cytotoxicity. After 24 and 48 h, MTT (Sigma-Aldrich Chemical, Warsaw, Poland) colorimetric assay was performed to evaluate cell viability as described previously [39]. The results of the MTT assay were expressed as the percentage of absorbance value obtained with the negative control.

Cytotoxicity assessment in direct contact of osteoblasts with the scaffolds was performed by live-dead double fluorescent staining. The MC3T3-E1 cells were seeded directly on the scaffold discs (2 mm thick and 8 mm in diameter) in 500 µL of a complete culture medium at a density of 3 × 10^5^ cells/mL. After 48 h of culture, osteoblasts were stained with Live-Dead Double Staining Kit (Sigma-Aldrich Chemical, Warsaw, Poland) in accordance with the manufacturer’s procedure. The kit is comprised of calcein-AM dye and propidium iodide dye, which stains viable and dead cells, respectively. Stained cells were observed using a confocal laser scanning microscope (CLSM, Olympus Fluoview equipped with FV1000, Olympus Polska Sp. z o. o., Warsaw, Poland).

#### 3.4.2. Cell Adhesion, Spreading, and Proliferation Assessment

Evaluation of cell adhesion, spreading, and proliferation was carried out by seeding MC3T3-1 cells directly on the scaffolds as well as by culturing the osteoblasts in 96-multiwell plate in 24 h extracts of the samples, prepared as described in Section 3.3. The MC3T3-E1 cells were seeded directly on the scaffold discs (2 mm thick and 5 mm in diameter) placed in the wells of 96-multiwell plate and in the PS wells of 96-multiwell plate in 100 μL of complete culture medium at a density of 8 × 10^4^ cells/mL. After 24 h culture of osteoblasts in PS wells, the culture medium was replaced with appropriate extracts. In the case of cell adhesion and the spreading experiment, on the second day, the cells were fixed with 3.7% (*v*/*v*) paraformaldehyde (Sigma-Aldrich Chemicals, Warsaw, Poland) for 10 min, permeabilized with 0.2% (*v*/*v*) Triton X-100 (Sigma-Aldrich Chemicals, Warsaw, Poland) for 20 min, and blocked with 1% (*w*/*v*) bovine serum albumin (BSA, Sigma-Aldrich Chemicals, Warsaw, Poland) for 30 min. Then, the cytoskeleton filaments were stained for 30 min at room temperature with AlexaFluor635-conjugated phallotoxin (Invitrogen, Carlsbad, California, USA). Counterstaining was conducted using 0.5 μg/mL DAPI (Sigma-Aldrich Chemicals, Warsaw, Poland). Stained cells were observed using CLSM. Spreading area of at least 80 individual cells was measured using ImageJ software version 1.52a (Wayne Rasband, National Institutes of Health, Bethesda, Maryland, USA)

The cell proliferation test was carried out for 6 days. Every second day of the experiment, half of the culture medium or appropriate extract was replaced with a fresh portion. On the first, third, and sixth day, osteoblasts were lysed, and the total cell number was determined based on the Lactate Dehydrogenase Activity Assay Kit (Sigma-Aldrich Chemicals, Warsaw, Poland) in accordance with the manufacturer’s protocol. The cell number was calculated from the calibration curve made for known concentrations of MC3T3-E1 cells. The doubling time for the cells was calculated using Doubling Time Computing software version 3.1.0.

#### 3.4.3. Evaluation of Osteogenic Differentiation

Evaluation of osteogenic differentiation was carried out by seeding mesenchymal stem cells directly on the scaffolds, as well as by culturing the cells in 96-multiwell plate in 24 h extracts of the samples, prepared as described in Section 3.3 but using osteogenic medium (Osteocyte Differentiation Tool, ATCC-LGC standards, Teddington, UK). The BMDSCs and ADSCs were seeded directly on the scaffold discs (2 mm thick and 8 mm in diameter) placed in the wells of 48-multiwell plate and in the PS wells of 48-multiwell plate in 500 µL of complete culture medium at a density 2 × 10^5^ cells/mL. After 24 h culture of the cells, osteogenic differentiation of the BMDSCs and ADSCs were induced by replacing the culture medium with osteogenic medium or appropriate extracts of the samples (prepared in osteogenic medium). Every third day, half of the medium/extract was replaced with a fresh portion. On the twenty-first day of the study, markers of the osteogenic differentiation (Col I, bALP and OC) were quantitatively evaluated in the cell lysates prepared as previously reported [40]. The Col I, bALP, and OC levels were measured using human-specific ELISA kits (Human Collagen alpha-1(I) chain ELISA Kit, EIAab, Wuhan, China; Human Bone Alkaline Phosphatase ELISA Kit, FineTest, Wuhan, China; Human Osteocalcin ELISA Kit, EIAab, Wuhan, China). The level of osteogenic markers was normalized to the total protein content, which was determined for each sample/well using a BCA Protein Assay Kit (Thermo Fisher Scientific, Waltham, Massachusetts, USA). The ELISA results were expressed as ng of osteogenic marker (Col I, bALP and OC) per mg of total cellular proteins.

In the case of cells cultured in the PS wells in the presence of the extracts, osteogenic differentiation was also visualized by IF staining of Col I and OC and by ARS staining of mineral deposited in ECM. For IF, the samples were fixed and permeabilized as described in Section 3.4.2. Then, the samples were incubated overnight at 4 °C with primary goat anticollagen I (Col1a1/Col1a2) polyclonal antibody (Abnova, Taoyuan City, Taiwan) and primary mouse anti-osteocalcin monoclonal antibody (Abcam, Cambridge, UK) prepared at a concentration of 10 µg/mL. Subsequently, the samples were washed with PBS and incubated for 1 h at room temperature with secondary Alexa-Fluor^®^647 donkey anti-goat IgG (H+L) antibody (Abcam, Cambridge, UK) and secondary Alexa-Fluor^®^488 donkey anti-mouse IgG (H+L) antibody (Abcam, Cambridge, UK) was prepared at a concentration of 2 µg/mL. Additionally, the cell nuclei were counterstained with the 0.5 µg/mL DAPI solution. Stained cells were visualized using CLSM. After IF staining of Col I and OC, the same samples were stained for 15 min at 37 °C with 2% (*w*/*v*) alizarin red solution (Sigma-Aldrich Chemicals, Warsaw, Poland) prepared in deionized water. Then, the staining solution was removed, and the cells were rinsed 10 times with PBS without calcium and magnesium ions. The mineral deposits in ECM were visualized by a stereoscopic and phase-contrast microscope (Olympus CKX53, Olympus Polska Sp. z o. o., Warsaw, Poland). Afterwards, the same samples were subjected to quantitative analysis to determine the exact concentrations of deposited mineral. Briefly, 200 µL of 10% acetic acid (Sigma-Aldrich Chemicals, Warsaw, Poland) were added to each well for 30 min in order to desorb calcium-bound ARS. Next, the cells were scraped from the plate and subjected to heating for 10 min at 85 °C. The cooled samples were centrifuged and 75 µL of 10% ammonium hydroxide (Avantor Performance Materials, Gliwice, Poland) were added to the supernatants to neutralize the pH. The OD values were detected at 405 nm (BioTek Synergy H4 Hybrid Microplate Reader, Winooski, Vermont, USA). Accurate amounts of deposited HA mineral were calculated from the calibration curve prepared for known concentrations of HA. Mineral deposition was normalized to total protein content and expressed as mg of mineral per mg of total cellular proteins. Unfortunately, quantification of mineral in the cells cultured directly on the scaffolds was impossible because the HA-based samples had the ability to bind ARS dye.

### 3.5. Statistical Analysis

All experiments were performed at least in triplicate (*n* = 3). The results were presented as mean values ± standard deviation (SD). Statistically significant differences among all groups (control and various scaffolds) (considered at *P* < 0.05) were determined by one-way ANOVA followed by Tukey’s test (GraphPad Prism 8.0.0 Software).

## 4. Patents

The method for the production of the scaffolds was claimed in the Polish patent application no P.426788.

## Figures and Tables

**Table 1:** Parameters obtained from the PXRD patterns: unit cell parameters a and c (nm) and average crystallite sizes determined from (002) reflection.

Nanopowder		HA	HA-Mg	HA-Zn
Unit cell parameters (nm) *	a	0.943	0.9425	0.9420
	c	0.6872	0.6853	0.6818
Crystal size (nm)		65 ± 5	58 ± 4	64 ± 6

* The error does not exceed 0.3%.

**Table 2:** Mg^2+^ and Zn^2+^ ion concentrations [µg/mL] in scaffold extracts prepared by 24 h incubation of the materials in culture medium.

Conc. (µg/mL)	Control Culture Medium	Chit/Aga/HA	Chit/Aga/HA-Mg	Chit/Aga/HA-Zn	Chit/Aga/HA-Mg/Zn
Mg^2+^	29.12 ± 3.14	16.90 ± 0.83 *^#$^	41.04 ± 1.09 *	21.36 ± 1.50 *^#$^	36.16 ± 2.67 *
Zn^2+^	0.16 ± 0.06	0.11 ± 0.02 ^$&^	0.12 ± 0.03	4.42 ± 0.25 *^#$^	0.64 ± 0.13 *^#^

* statistically significant results as compared to the control medium; ^#^ statistically significant results as compared to chit/aga/HA-Mg; ^$^ statistically significant results as compared to chit/aga/HA-Mg/Zn; ^&^ statistically significant results as compared to chit/aga/HA-Zn (*P* < 0.05, one-way ANOVA followed by Tukey’s test).

## Abbreviations

ADSCs	Adipose tissue-derived mesenchymal stem cells
bALP	Bone alkaline phosphatase
BMDSCs	Human bone marrow-derived stem cells
BSA	Bovine serum albumin
CLSM	Confocal laser scanning microscope
Col I	Type I collagen
DT	Doubling time
ECM	Extracellular matrix
ELISA	Enzyme-linked immunosorbent assay
FBS	Fetal bovine serum
FT-IR	Fourier-transform infrared spectroscopy
HA	Hydroxyapatite
ICP-OES	Inductively coupled plasma spectrometry
IF	Immunofluorescence
IGF-1	Insulin-like growth factor 1
MC3T3-E1	Mouse calvarial preosteoblast cell line
MSCs	Mesenchymal stem cells
OC	Osteocalcin
PS	Polystyrene
PXRD	Powder X-ray diffraction
Runx-2	Runt-related transcription factor 2
TEM	Transmission electron microscope
VEGF	Vascular endothelial growth factor
